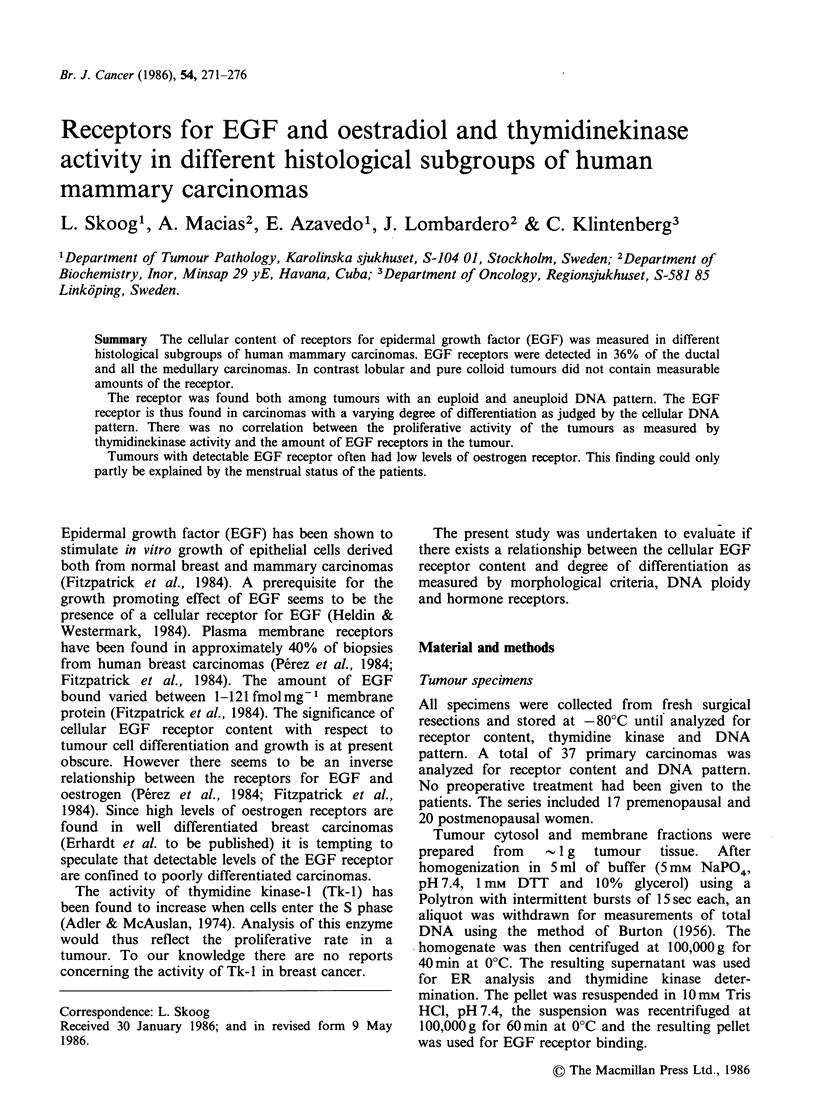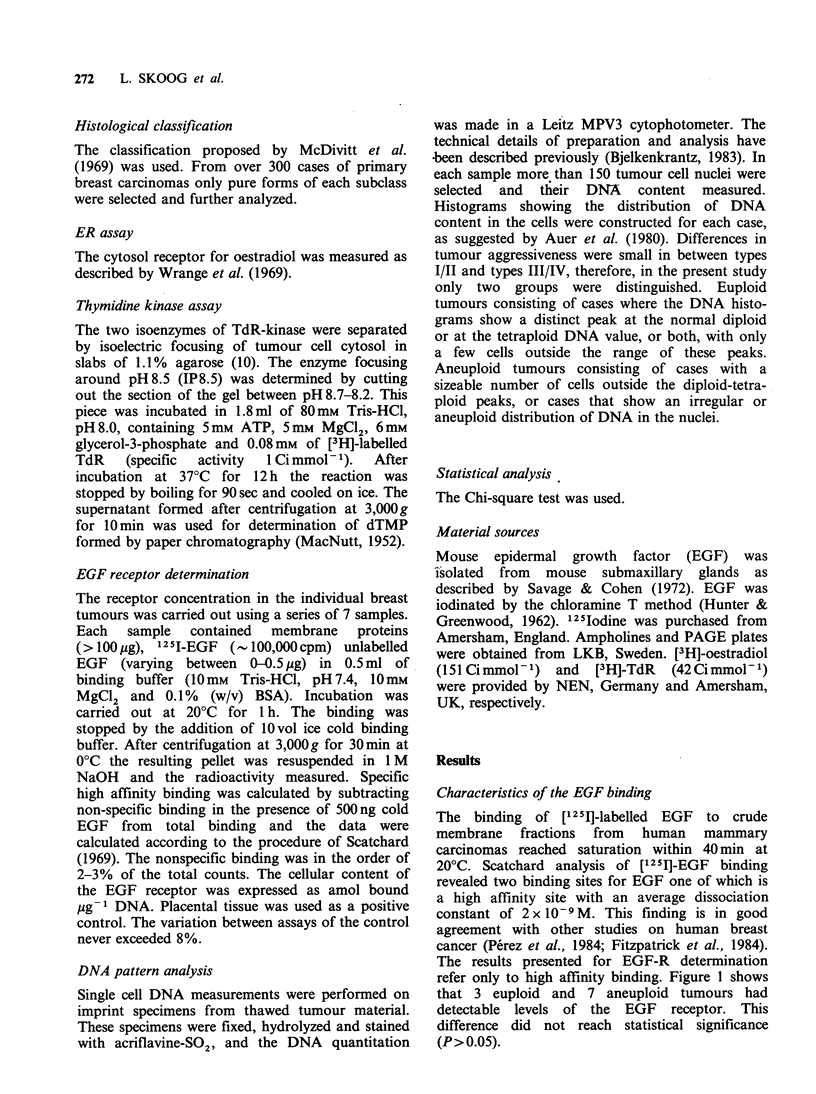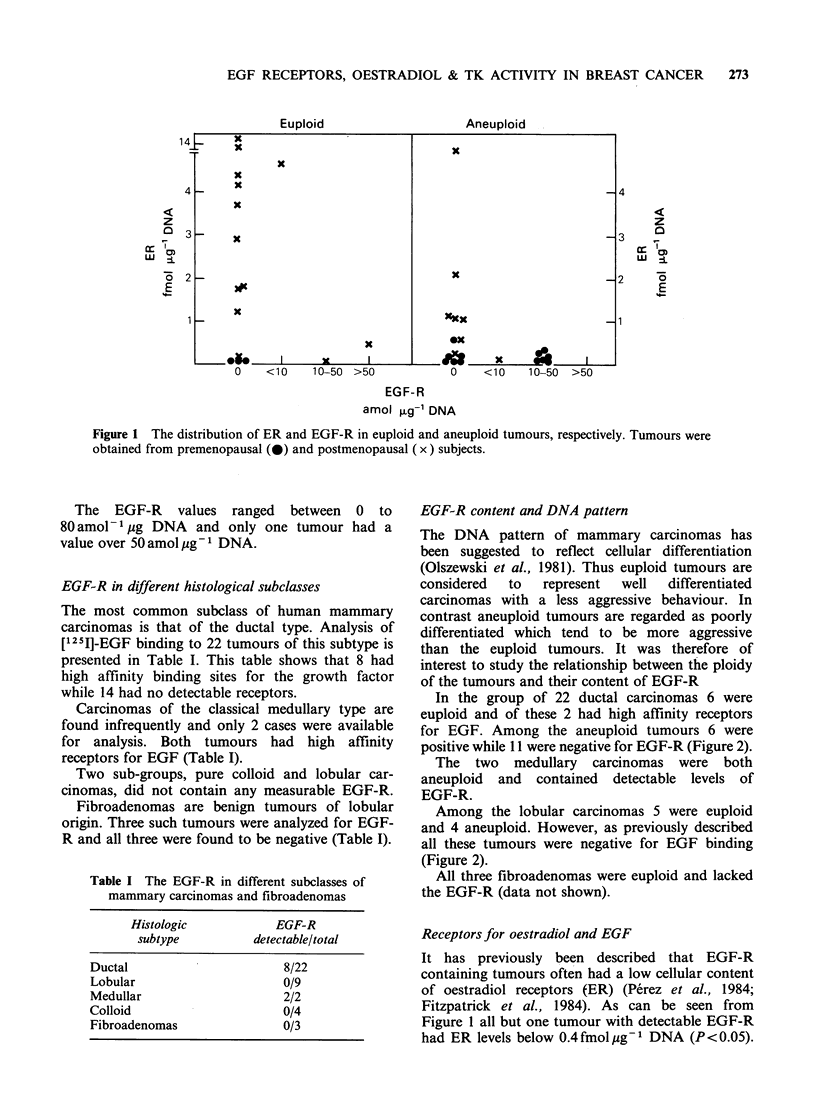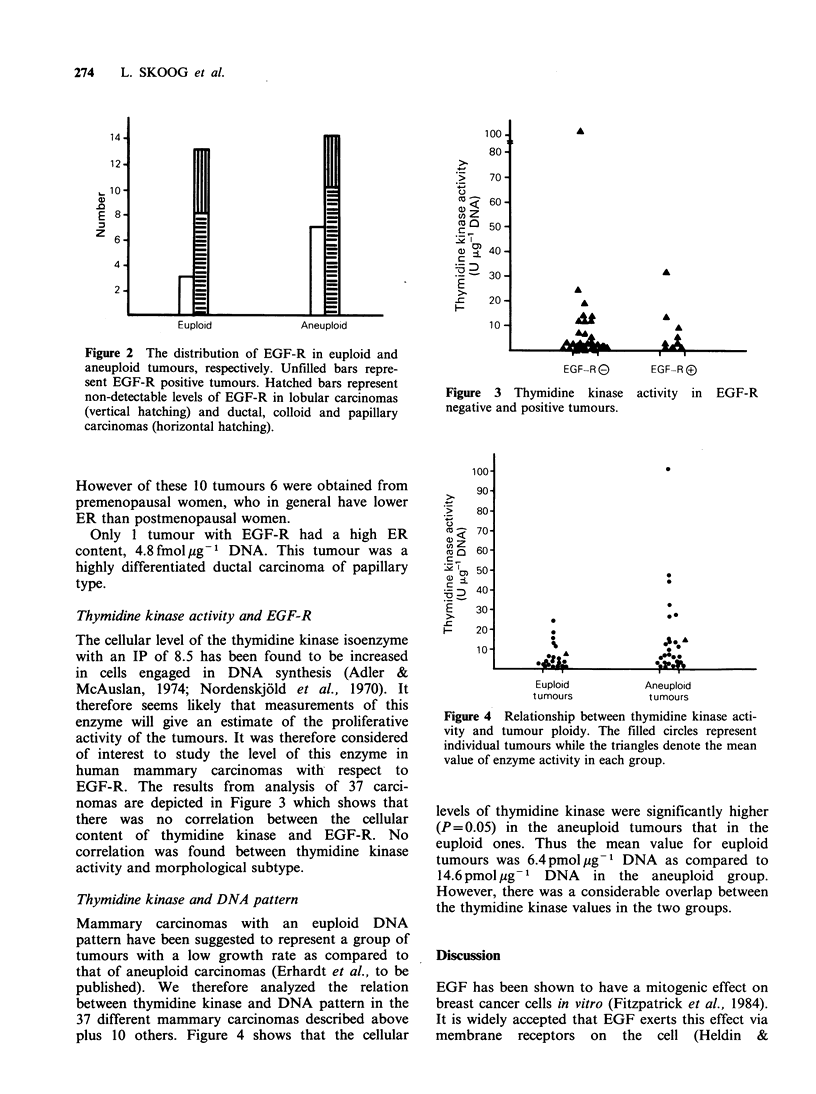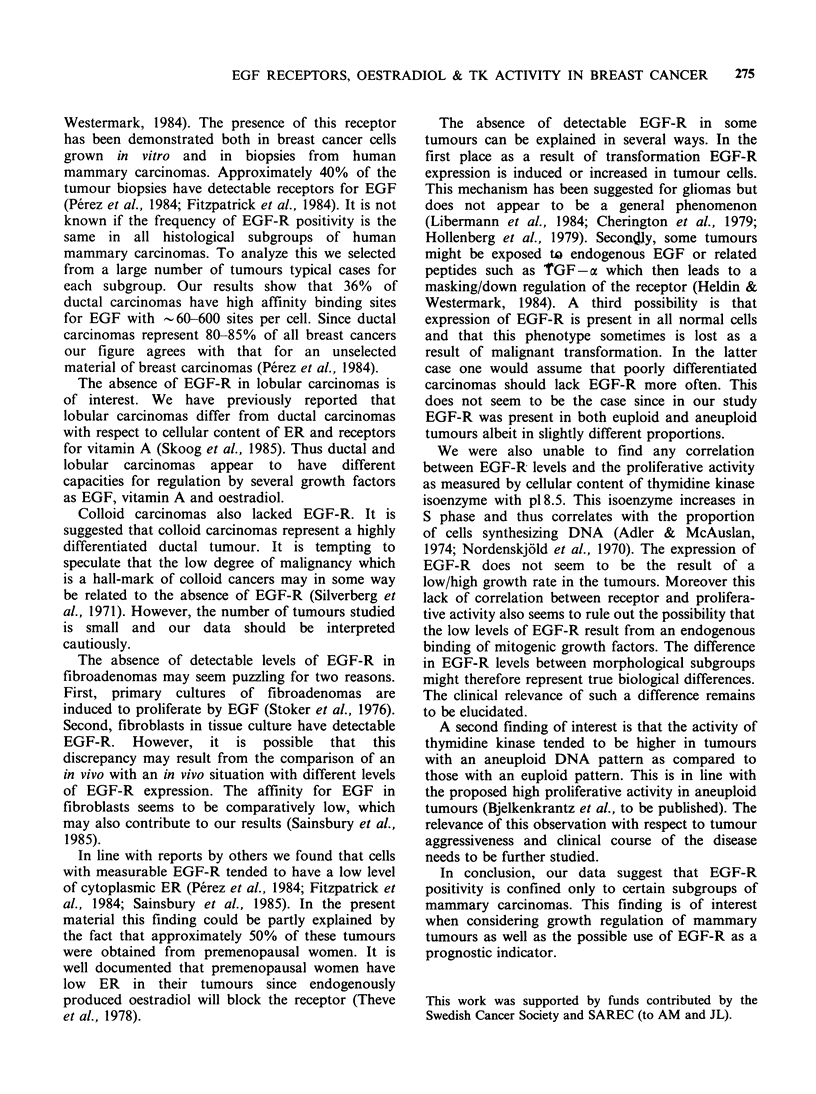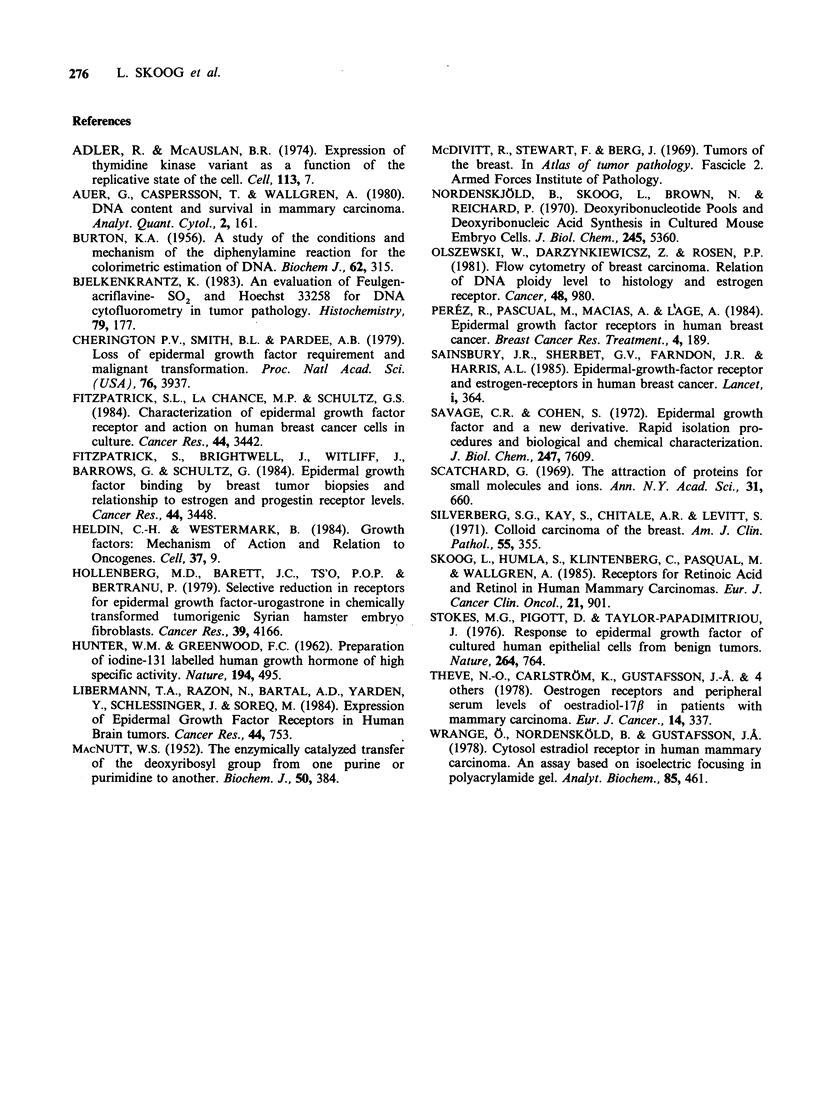# Receptors for EGF and oestradiol and thymidinekinase activity in different histological subgroups of human mammary carcinomas.

**DOI:** 10.1038/bjc.1986.173

**Published:** 1986-08

**Authors:** L. Skoog, A. Macias, E. Azavedo, J. Lombardero, C. Klintenberg

## Abstract

The cellular content of receptors for epidermal growth factor (EGF) was measured in different histological subgroups of human mammary carcinomas. EGF receptors were detected in 36% of the ductal and all the medullary carcinomas. In contrast lobular and pure colloid tumours did not contain measurable amounts of the receptor. The receptor was found both among tumours with an euploid and aneuploid DNA pattern. The EGF receptor is thus found in carcinomas with a varying degree of differentiation as judged by the cellular DNA pattern. There was no correlation between the proliferative activity of the tumours as measured by thymidinekinase activity and the amount of EGF receptors in the tumour. Tumours with detectable EGF receptor often had low levels of oestrogen receptor. This finding could only partly be explained by the menstrual status of the patients.


*Br. J. Cancer* (1986), **54**, 271–276

### Receptors for EGF and oestradiol and thymidinekinase activity in different histological subgroups of human mammary carcinomas

L. Skoog[1], A. Macias[2], E. Azavedo[1], J. Lombardero[2] & C. Klintenberg[3]

[1]*Department of Tumour Pathology, Karolinska sjukhuset, S-104 01, Stockholm, Sweden;* [2]*Department of Biochemistry, Inor, Minsap 29 yE, Havana, Cuba;* [3]*Department of Oncology, Regionsjukhuset, S-581 85 Linköping, Sweden.*

**Summary** The cellular content of receptors for epidermal growth factor (EGF) was measured in different histological subgroups of human mammary carcinomas. EGF receptors were detected in 36% of the ductal and all the medullary carcinomas. In contrast lobular and pure colloid tumours did not contain measurable amounts of the receptor.

The receptor was found both among tumours with an euploid and aneuploid DNA pattern. The EGF receptor is thus found in carcinomas with a varying degree of differentiation as judged by the cellular DNA pattern. There was no correlation between the proliferative activity of the tumours as measured by thymidinekinase activity and the amount of EGF receptors in the tumour.

Tumours with detectable EGF receptor often had low levels of oestrogen receptor. This finding could only partly be explained by the menstrual status of the patients.

Epidermal growth factor (EGF) has been shown to stimulate *in vitro* growth of epithelial cells derived both from normal breast and mammary carcinomas (Fitzpatrick *et al.*, 1984). A prerequisite for the growth promoting effect of EGF seems to be the presence of a cellular receptor for EGF (Heldin & Westermark, 1984). Plasma membrane receptors have been found in approximately 40% of biopsies from human breast carcinomas (Pérez *et al.*, 1984; Fitzpatrick *et al.*, 1984). The amount of EGF bound varied between 1–121 fmol $mg^{-1}$ membrane protein (Fitzpatrick *et al.*, 1984). The significance of cellular EGF receptor content with respect to tumour cell differentiation and growth is at present obscure. However there seems to be an inverse relationship between the receptors for EGF and oestrogen (Pérez *et al.*, 1984; Fitzpatrick *et al.*, 1984). Since high levels of oestrogen receptors are found in well differentiated breast carcinomas (Erhardt *et al.* to be published) it is tempting to speculate that detectable levels of the EGF receptor are confined to poorly differentiated carcinomas.

The activity of thymidine kinase-1 (Tk-1) has been found to increase when cells enter the S phase (Adler & McAuslan, 1974). Analysis of this enzyme would thus reflect the proliferative rate in a tumour. To our knowledge there are no reports concerning the activity of Tk-1 in breast cancer.

Correspondence: L. Skoog
Received 30 January 1986; and in revised form 9 May 1986.

The present study was undertaken to evaluate if there exists a relationship between the cellular EGF receptor content and degree of differentiation as measured by morphological criteria, DNA ploidy and hormone receptors.

### Material and methods

### *Tumour specimens*

All specimens were collected from fresh surgical resections and stored at −80°C until analyzed for receptor content, thymidine kinase and DNA pattern. A total of 37 primary carcinomas was analyzed for receptor content and DNA pattern. No preoperative treatment had been given to the patients. The series included 17 premenopausal and 20 postmenopausal women.

Tumour cytosol and membrane fractions were prepared from ~1 g tumour tissue. After homogenization in 5 ml of buffer (5 mM $NaPO_4$, pH 7.4, 1 mM DTT and 10% glycerol) using a Polytron with intermittent bursts of 15 sec each, an aliquot was withdrawn for measurements of total DNA using the method of Burton (1956). The homogenate was then centrifuged at 100,000 g for 40 min at 0°C. The resulting supernatant was used for ER analysis and thymidine kinase determination. The pellet was resuspended in 10 mM Tris HCl, pH 7.4, the suspension was recentrifuged at 100,000 g for 60 min at 0°C and the resulting pellet was used for EGF receptor binding.

© The Macmillan Press Ltd., 1986

*Histological classification*

The classification proposed by McDivitt *et al.* (1969) was used. From over 300 cases of primary breast carcinomas only pure forms of each subclass were selected and further analyzed.

*ER assay*

The cytosol receptor for oestradiol was measured as described by Wrange *et al.* (1969).

*Thymidine kinase assay*

The two isoenzymes of TdR-kinase were separated by isoelectric focusing of tumour cell cytosol in slabs of 1.1% agarose (10). The enzyme focusing around pH 8.5 (IP8.5) was determined by cutting out the section of the gel between pH 8.7–8.2. This piece was incubated in 1.8 ml of 80 mM Tris-HCl, pH 8.0, containing 5 mM ATP, 5 mM $MgCl_2$, 6 mM glycerol-3-phosphate and 0.08 mM of [$^3$H]-labelled TdR (specific activity 1 Ci mmol$^{-1}$). After incubation at 37°C for 12 h the reaction was stopped by boiling for 90 sec and cooled on ice. The supernatant formed after centrifugation at 3,000 *g* for 10 min was used for determination of dTMP formed by paper chromatography (MacNutt, 1952).

*EGF receptor determination*

The receptor concentration in the individual breast tumours was carried out using a series of 7 samples. Each sample contained membrane proteins (>100 μg), $^{125}$I-EGF (~100,000 cpm) unlabelled EGF (varying between 0–0.5 μg) in 0.5 ml of binding buffer (10 mM Tris-HCl, pH 7.4, 10 mM $MgCl_2$ and 0.1% (w/v) BSA). Incubation was carried out at 20°C for 1 h. The binding was stopped by the addition of 10 vol ice cold binding buffer. After centrifugation at 3,000 *g* for 30 min at 0°C the resulting pellet was resuspended in 1 M NaOH and the radioactivity measured. Specific high affinity binding was calculated by subtracting non-specific binding in the presence of 500 ng cold EGF from total binding and the data were calculated according to the procedure of Scatchard (1969). The nonspecific binding was in the order of 2–3% of the total counts. The cellular content of the EGF receptor was expressed as amol bound μg$^{-1}$ DNA. Placental tissue was used as a positive control. The variation between assays of the control never exceeded 8%.

*DNA pattern analysis*

Single cell DNA measurements were performed on imprint specimens from thawed tumour material. These specimens were fixed, hydrolyzed and stained with acriflavine-$SO_2$, and the DNA quantitation was made in a Leitz MPV3 cytophotometer. The technical details of preparation and analysis have been described previously (Bjelkenkrantz, 1983). In each sample more than 150 tumour cell nuclei were selected and their DNA content measured. Histograms showing the distribution of DNA content in the cells were constructed for each case, as suggested by Auer *et al.* (1980). Differences in tumour aggressiveness were small in between types I/II and types III/IV, therefore, in the present study only two groups were distinguished. Euploid tumours consisting of cases where the DNA histograms show a distinct peak at the normal diploid or at the tetraploid DNA value, or both, with only a few cells outside the range of these peaks. Aneuploid tumours consisting of cases with a sizeable number of cells outside the diploid-tetraploid peaks, or cases that show an irregular or aneuploid distribution of DNA in the nuclei.

*Statistical analysis*

The Chi-square test was used.

*Material sources*

Mouse epidermal growth factor (EGF) was isolated from mouse submaxillary glands as described by Savage & Cohen (1972). EGF was iodinated by the chloramine T method (Hunter & Greenwood, 1962). $^{125}$Iodine was purchased from Amersham, England. Ampholines and PAGE plates were obtained from LKB, Sweden. [$^3$H]-oestradiol (151 Ci mmol$^{-1}$) and [$^3$H]-TdR (42 Ci mmol$^{-1}$) were provided by NEN, Germany and Amersham, UK, respectively.

### Results

*Characteristics of the EGF binding*

The binding of [$^{125}$I]-labelled EGF to crude membrane fractions from human mammary carcinomas reached saturation within 40 min at 20°C. Scatchard analysis of [$^{125}$I]-EGF binding revealed two binding sites for EGF one of which is a high affinity site with an average dissociation constant of $2 \times 10^{-9}$ M. This finding is in good agreement with other studies on human breast cancer (Pérez *et al.*, 1984; Fitzpatrick *et al.*, 1984). The results presented for EGF-R determination refer only to high affinity binding. Figure 1 shows that 3 euploid and 7 aneuploid tumours had detectable levels of the EGF receptor. This difference did not reach statistical significance ($P > 0.05$).

**Figure 1** The distribution of ER and EGF-R in euploid and aneuploid tumours, respectively. Tumours were obtained from premenopausal (●) and postmenopausal (×) subjects.

The EGF-R values ranged between 0 to 80 amol$^{-1}$ $\mu$g DNA and only one tumour had a value over 50 amol $\mu g^{-1}$ DNA.

*EGF-R in different histological subclasses*

The most common subclass of human mammary carcinomas is that of the ductal type. Analysis of [$^{125}$I]-EGF binding to 22 tumours of this subtype is presented in Table I. This table shows that 8 had high affinity binding sites for the growth factor while 14 had no detectable receptors.

Carcinomas of the classical medullary type are found infrequently and only 2 cases were available for analysis. Both tumours had high affinity receptors for EGF (Table I).

Two sub-groups, pure colloid and lobular carcinomas, did not contain any measurable EGF-R.

Fibroadenomas are benign tumours of lobular origin. Three such tumours were analyzed for EGF-R and all three were found to be negative (Table I).

**Table I** The EGF-R in different subclasses of mammary carcinomas and fibroadenomas

| *Histologic subtype* | *EGF-R detectable/total* |
|---|---|
| Ductal | 8/22 |
| Lobular | 0/9 |
| Medullar | 2/2 |
| Colloid | 0/4 |
| Fibroadenomas | 0/3 |

*EGF-R content and DNA pattern*

The DNA pattern of mammary carcinomas has been suggested to reflect cellular differentiation (Olszewski *et al.*, 1981). Thus euploid tumours are considered to represent well differentiated carcinomas with a less aggressive behaviour. In contrast aneuploid tumours are regarded as poorly differentiated which tend to be more aggressive than the euploid tumours. It was therefore of interest to study the relationship between the ploidy of the tumours and their content of EGF-R

In the group of 22 ductal carcinomas 6 were euploid and of these 2 had high affinity receptors for EGF. Among the aneuploid tumours 6 were positive while 11 were negative for EGF-R (Figure 2).

The two medullary carcinomas were both aneuploid and contained detectable levels of EGF-R.

Among the lobular carcinomas 5 were euploid and 4 aneuploid. However, as previously described all these tumours were negative for EGF binding (Figure 2).

All three fibroadenomas were euploid and lacked the EGF-R (data not shown).

*Receptors for oestradiol and EGF*

It has previously been described that EGF-R containing tumours often had a low cellular content of oestradiol receptors (ER) (Pérez *et al.*, 1984; Fitzpatrick *et al.*, 1984). As can be seen from Figure 1 all but one tumour with detectable EGF-R had ER levels below 0.4 fmol $\mu g^{-1}$ DNA ($P<0.05$).

**Figure 2** The distribution of EGF-R in euploid and aneuploid tumours, respectively. Unfilled bars represent EGF-R positive tumours. Hatched bars represent non-detectable levels of EGF-R in lobular carcinomas (vertical hatching) and ductal, colloid and papillary carcinomas (horizontal hatching).

**Figure 3** Thymidine kinase activity in EGF-R negative and positive tumours.

However of these 10 tumours 6 were obtained from premenopausal women, who in general have lower ER than postmenopausal women.

Only 1 tumour with EGF-R had a high ER content, 4.8 fmol μg$^{-1}$ DNA. This tumour was a highly differentiated ductal carcinoma of papillary type.

*Thymidine kinase activity and EGF-R*

The cellular level of the thymidine kinase isoenzyme with an IP of 8.5 has been found to be increased in cells engaged in DNA synthesis (Adler & McAuslan, 1974; Nordenskjöld *et al.*, 1970). It therefore seems likely that measurements of this enzyme will give an estimate of the proliferative activity of the tumours. It was therefore considered of interest to study the level of this enzyme in human mammary carcinomas with respect to EGF-R. The results from analysis of 37 carcinomas are depicted in Figure 3 which shows that there was no correlation between the cellular content of thymidine kinase and EGF-R. No correlation was found between thymidine kinase activity and morphological subtype.

*Thymidine kinase and DNA pattern*

Mammary carcinomas with an euploid DNA pattern have been suggested to represent a group of tumours with a low growth rate as compared to that of aneuploid carcinomas (Erhardt *et al.*, to be published). We therefore analyzed the relation between thymidine kinase and DNA pattern in the 37 different mammary carcinomas described above plus 10 others. Figure 4 shows that the cellular

**Figure 4** Relationship between thymidine kinase activity and tumour ploidy. The filled circles represent individual tumours while the triangles denote the mean value of enzyme activity in each group.

levels of thymidine kinase were significantly higher ($P = 0.05$) in the aneuploid tumours that in the euploid ones. Thus the mean value for euploid tumours was 6.4 pmol μg$^{-1}$ DNA as compared to 14.6 pmol μg$^{-1}$ DNA in the aneuploid group. However, there was a considerable overlap between the thymidine kinase values in the two groups.

### Discussion

EGF has been shown to have a mitogenic effect on breast cancer cells *in vitro* (Fitzpatrick *et al.*, 1984). It is widely accepted that EGF exerts this effect via membrane receptors on the cell (Heldin &

Westermark, 1984). The presence of this receptor has been demonstrated both in breast cancer cells grown *in vitro* and in biopsies from human mammary carcinomas. Approximately 40% of the tumour biopsies have detectable receptors for EGF (Pérez *et al.*, 1984; Fitzpatrick *et al.*, 1984). It is not known if the frequency of EGF-R positivity is the same in all histological subgroups of human mammary carcinomas. To analyze this we selected from a large number of tumours typical cases for each subgroup. Our results show that 36% of ductal carcinomas have high affinity binding sites for EGF with ~60–600 sites per cell. Since ductal carcinomas represent 80–85% of all breast cancers our figure agrees with that for an unselected material of breast carcinomas (Pérez *et al.*, 1984).

The absence of EGF-R in lobular carcinomas is of interest. We have previously reported that lobular carcinomas differ from ductal carcinomas with respect to cellular content of ER and receptors for vitamin A (Skoog *et al.*, 1985). Thus ductal and lobular carcinomas appear to have different capacities for regulation by several growth factors as EGF, vitamin A and oestradiol.

Colloid carcinomas also lacked EGF-R. It is suggested that colloid carcinomas represent a highly differentiated ductal tumour. It is tempting to speculate that the low degree of malignancy which is a hall-mark of colloid cancers may in some way be related to the absence of EGF-R (Silverberg *et al.*, 1971). However, the number of tumours studied is small and our data should be interpreted cautiously.

The absence of detectable levels of EGF-R in fibroadenomas may seem puzzling for two reasons. First, primary cultures of fibroadenomas are induced to proliferate by EGF (Stoker *et al.*, 1976). Second, fibroblasts in tissue culture have detectable EGF-R. However, it is possible that this discrepancy may result from the comparison of an *in vivo* with an *in vivo* situation with different levels of EGF-R expression. The affinity for EGF in fibroblasts seems to be comparatively low, which may also contribute to our results (Sainsbury *et al.*, 1985).

In line with reports by others we found that cells with measurable EGF-R tended to have a low level of cytoplasmic ER (Pérez *et al.*, 1984; Fitzpatrick *et al.*, 1984; Sainsbury *et al.*, 1985). In the present material this finding could be partly explained by the fact that approximately 50% of these tumours were obtained from premenopausal women. It is well documented that premenopausal women have low ER in their tumours since endogenously produced oestradiol will block the receptor (Theve *et al.*, 1978).

The absence of detectable EGF-R in some tumours can be explained in several ways. In the first place as a result of transformation EGF-R expression is induced or increased in tumour cells. This mechanism has been suggested for gliomas but does not appear to be a general phenomenon (Libermann *et al.*, 1984; Cherington *et al.*, 1979; Hollenberg *et al.*, 1979). Secondly, some tumours might be exposed to endogenous EGF or related peptides such as TGF$-\alpha$ which then leads to a masking/down regulation of the receptor (Heldin & Westermark, 1984). A third possibility is that expression of EGF-R is present in all normal cells and that this phenotype sometimes is lost as a result of malignant transformation. In the latter case one would assume that poorly differentiated carcinomas should lack EGF-R more often. This does not seem to be the case since in our study EGF-R was present in both euploid and aneuploid tumours albeit in slightly different proportions.

We were also unable to find any correlation between EGF-R levels and the proliferative activity as measured by cellular content of thymidine kinase isoenzyme with pI 8.5. This isoenzyme increases in S phase and thus correlates with the proportion of cells synthesizing DNA (Adler & McAuslan, 1974; Nordenskjöld *et al.*, 1970). The expression of EGF-R does not seem to be the result of a low/high growth rate in the tumours. Moreover this lack of correlation between receptor and proliferative activity also seems to rule out the possibility that the low levels of EGF-R result from an endogenous binding of mitogenic growth factors. The difference in EGF-R levels between morphological subgroups might therefore represent true biological differences. The clinical relevance of such a difference remains to be elucidated.

A second finding of interest is that the activity of thymidine kinase tended to be higher in tumours with an aneuploid DNA pattern as compared to those with an euploid pattern. This is in line with the proposed high proliferative activity in aneuploid tumours (Bjelkenkrantz *et al.*, to be published). The relevance of this observation with respect to tumour aggressiveness and clinical course of the disease needs to be further studied.

In conclusion, our data suggest that EGF-R positivity is confined only to certain subgroups of mammary carcinomas. This finding is of interest when considering growth regulation of mammary tumours as well as the possible use of EGF-R as a prognostic indicator.

This work was supported by funds contributed by the Swedish Cancer Society and SAREC (to AM and JL).

### References

ADLER, R. & McAUSLAN, B.R. (1974). Expression of thymidine kinase variant as a function of the replicative state of the cell. *Cell*, **113**, 7.

AUER, G., CASPERSSON, T. & WALLGREN, A. (1980). DNA content and survival in mammary carcinoma. *Analyt. Quant. Cytol.*, **2**, 161.

BURTON, K.A. (1956). A study of the conditions and mechanism of the diphenylamine reaction for the colorimetric estimation of DNA. *Biochem J.*, **62**, 315.

BJELKENKRANTZ, K. (1983). An evaluation of Feulgen-acriflavine- $SO_2$ and Hoechst 33258 for DNA cytofluorometry in tumor pathology. *Histochemistry*, **79**, 177.

CHERINGTON P.V., SMITH, B.L. & PARDEE, A.B. (1979). Loss of epidermal growth factor requirement and malignant transformation. *Proc. Natl Acad. Sci. (USA)*, **76**, 3937.

FITZPATRICK, S.L., LA CHANCE, M.P. & SCHULTZ, G.S. (1984). Characterization of epidermal growth factor receptor and action on human breast cancer cells in culture. *Cancer Res.*, **44**, 3442.

FITZPATRICK, S., BRIGHTWELL, J., WITLIFF, J., BARROWS, G. & SCHULTZ, G. (1984). Epidermal growth factor binding by breast tumor biopsies and relationship to estrogen and progestin receptor levels. *Cancer Res.*, **44**, 3448.

HELDIN, C.-H. & WESTERMARK, B. (1984). Growth factors: Mechanism of Action and Relation to Oncogenes. *Cell*, **37**, 9.

HOLLENBERG, M.D., BARETT, J.C., TS'O, P.O.P. & BERTRANU, P. (1979). Selective reduction in receptors for epidermal growth factor-urogastrone in chemically transformed tumorigenic Syrian hamster embryo fibroblasts. *Cancer Res.*, **39**, 4166.

HUNTER, W.M. & GREENWOOD, F.C. (1962). Preparation of iodine-131 labelled human growth hormone of high specific activity. *Nature*, **194**, 495.

LIBERMANN, T.A., RAZON, N., BARTAL, A.D., YARDEN, Y., SCHLESSINGER, J. & SOREQ, M. (1984). Expression of Epidermal Growth Factor Receptors in Human Brain tumors. *Cancer Res.*, **44**, 753.

MacNUTT, W.S. (1952). The enzymically catalyzed transfer of the deoxyribosyl group from one purine or purimidine to another. *Biochem. J.*, **50**, 384.

McDIVITT, R., STEWART, F. & BERG, J. (1969). Tumors of the breast. In *Atlas of tumor pathology*. Fascicle 2. Armed Forces Institute of Pathology.

NORDENSKJÖLD, B., SKOOG, L., BROWN, N. & REICHARD, P. (1970). Deoxyribonucleotide Pools and Deoxyribonucleic Acid Synthesis in Cultured Mouse Embryo Cells. *J. Biol. Chem.*, **245**, 5360.

OLSZEWSKI, W., DARZYNKIEWICSZ, Z. & ROSEN, P.P. (1981). Flow cytometry of breast carcinoma. Relation of DNA ploidy level to histology and estrogen receptor. *Cancer*, **48**, 980.

PERÉZ, R., PASCUAL, M., MACIAS, A. & L'AGE, A. (1984). Epidermal growth factor receptors in human breast cancer. *Breast Cancer Res. Treatment.*, **4**, 189.

SAINSBURY, J.R., SHERBET, G.V., FARNDON, J.R. & HARRIS, A.L. (1985). Epidermal-growth-factor receptor and estrogen-receptors in human breast cancer. *Lancet*, **i**, 364.

SAVAGE, C.R. & COHEN, S. (1972). Epidermal growth factor and a new derivative. Rapid isolation procedures and biological and chemical characterization. *J. Biol. Chem.*, **247**, 7609.

SCATCHARD, G. (1969). The attraction of proteins for small molecules and ions. *Ann. N.Y. Acad. Sci.*, **31**, 660.

SILVERBERG, S.G., KAY, S., CHITALE, A.R. & LEVITT, S. (1971). Colloid carcinoma of the breast. *Am. J. Clin. Pathol.*, **55**, 355.

SKOOG, L., HUMLA, S., KLINTENBERG, C., PASQUAL, M. & WALLGREN, A. (1985). Receptors for Retinoic Acid and Retinol in Human Mammary Carcinomas. *Eur. J. Cancer Clin. Oncol.*, **21**, 901.

STOKES, M.G., PIGOTT, D. & TAYLOR-PAPADIMITRIOU, J. (1976). Response to epidermal growth factor of cultured human epithelial cells from benign tumors. *Nature*, **264**, 764.

THEVE, N.-O., CARLSTRÖM, K., GUSTAFSSON, J.-Å. & 4 others (1978). Oestrogen receptors and peripheral serum levels of oestradiol-17$\beta$ in patients with mammary carcinoma. *Eur. J. Cancer.*, **14**, 337.

WRANGE, Ö., NORDENSKÖLD, B. & GUSTAFSSON, J.Å. (1978). Cytosol estradiol receptor in human mammary carcinoma. An assay based on isoelectric focusing in polyacrylamide gel. *Analyt. Biochem.*, **85**, 461.